# Effect of dietary soybean oil inclusion on liver-related transcription factors in a pig model for metabolic diseases

**DOI:** 10.1038/s41598-022-14069-1

**Published:** 2022-06-20

**Authors:** Simara Larissa Fanalli, Bruna Pereira Martins da Silva, Julia Dezen Gomes, Fernanda Nery Ciconello, Vivian Vezzoni de Almeida, Felipe André Oliveira Freitas, Gabriel Costa Monteiro Moreira, Bárbara Silva-Vignato, Juliana Afonso, James Reecy, James Koltes, Dawn Koltes, Luciana Correia Almeida Regitano, Júlio Cesar de Carvalho Baileiro, Luciana Freitas, Luiz Lehmann Coutinho, Heidge Fukumasu, Severino Matias de Alencar, Albino Luchiari Filho, Aline Silva Mello Cesar

**Affiliations:** 1 Faculty of Animal Science and Food Engineering, University of São Paulo, Campus Fernando Costa, Avenue Duque de Caxias Norte 225, Pirassununga, São Paulo 13635-900 Brazil; 2 Luiz de Queiroz College of Agriculture, University of São Paulo, Avenue Pádua Dias 11, Piracicaba, São Paulo 13418-900 Brazil; 3 College of Veterinary Medicine and Animal Science, Federal University of Goiás, Nova Veneza, km 8, Campus Samambaia, Goiânia, Goiás 74690-900 Brazil; 4 University of Liège, GIGA Medical Genomics, Unit of Animal Genomics, Quartier Hôpital, Avenue de l’Hôpital, 11, 4000 Liège, Belgium; 5 Embrapa Pecuária Sudeste, Km 234 s/nº, São Carlos, São Paulo 13560-970 Brazil; 6 Department of Animal Science, College of Agriculture and Life Sciences, Iowa State University, 1221, Kildee Hall, Ames, IA 50011-3150 USA; 7 College of Veterinary Medicine and Animal Science, University of São Paulo, Duque de Caxias Norte, 225, Pirassununga, São Paulo 13.635-900 Brazil; 8 DB Genética de Suínos, Avenue Juscelino Kubitschek de Oliveira, 2094, Patos de Minas, MG 38.706-000 Brazil

**Keywords:** Genetics, Functional genomics, Gene expression profiling

## Abstract

Dietary fatty acids (FA) are components of the lipids, which contribute to membrane structure, energy input, and biological functions related to cellular signaling and transcriptome regulation. However, the consumers still associate dietary FA with fat deposition and increased occurrence of metabolic diseases such as obesity and atherosclerosis. Previous studies already demonstrated that some fatty acids are linked with inflammatory response, preventing metabolic diseases. To better understand the role of dietary FA on metabolic diseases, for the first time, a study to identify key transcription factors (TF) involved in lipid metabolism and inflammatory response by transcriptome analysis from liver samples of animal models was performed. The key TF were identified by functional enrichment analysis from the list of differentially expressed genes identified in liver samples between 35 pigs fed with 1.5% or 3.0% soybean oil. The functional enrichment analysis detected TF linked to lipid homeostasis and inflammatory response, such as *RXRA*, *EGFR*, and *SREBP2* precursor. These findings demonstrated that key TF related to lipid metabolism could be modulated by dietary inclusion of soybean oil. It could contribute to nutrigenomics research field that aims to elucidate dietary interventions in animal and human health, as well as to drive food technology and science.

## Introduction

Fatty acids are the main compound of lipids, which are a class of molecules present in animals and vegetal cell types. The main vegetal sources of dietary fatty acids in animal and human nutrition are soybean, canola, sunflower, corn, and flaxseed oil^1^. Its oils are rich sources of unsaturated fatty acids, such as monounsaturated (MUFA) and polyunsaturated (PUFA) fatty acids, which are previously associated with the prevention of health disorders because of their anti-inflammatory effects and cell membrane properties and structure^2^. Essential FA, mainly polyunsaturated fatty acids (PUFA), may modulate gene expression in diverse biological processes thought regulating transcription factors (TF), including peroxisome proliferator receptors *(PPAR)*, liver X receptors *(LXR)*, and sterol regulatory element-binding proteins (*SREBP*)^3^.

Soybean oil is considered an excellent source of unsaturated FA, such as linoleic acid (LA, C18:2 n-6), alpha-linoleic acid (ALA, C18:3 n-3), oleic acid (OA, C18:1 n-9), and palmitoleic acid (C16:1), which participate in lipid metabolism, inflammatory response, and cholesterol synthesis. Special attention has been focused on PUFA, specifically LA and ALA, given their functions of maintaining cell membranes under normal conditions, as well as their brain functions and the transmission of nerve impulses^2,4^. An increased proportion of omega (n)-6:n-3 PUFA, which is observed typically in Western diets, leads to a wide range of pathologies, such as cardiovascular, inflammatory, autoimmune, and diabetic diseases. On the other hand, a low ratio of n-6: n-3 PUFA in the diet may be linked to cholesterol reduction and prevention of cardiovascular diseases^5,6^.

The immune system is defined as the set of cells, tissues, and molecules that act as intermediates in defense of the organism against infections, conferring immunity, which comprises two functional systems: innate and adaptive^7^. In general, the main difference between these two immune systems lies in the mechanisms and receptors used for immunological recognition^8^. Immunonutrition refers to circumstances where feeding specific nutrients present a potential to modulate the activity of the immune system. This concept can be applied to any situation where a source of nutrients is used to modify the immune system or immune responses^9^.

It is already known that macro and micronutrients have immunomodulatory action (immune nutrients), which have been studied in intensive production systems, such as poultry and swine where animals are in contact with a wide range of potentially pathogenic microorganisms. The manifestation of infectious or pathological diseases has a nutritional cost to the animals since there are studies showing that animals raised in low sanitary environments grow slower and consume less food than animals raised in cleaner environments. This occurs because some nutrients that would be directed to the growth of the animal can be redirected to aid the response to the animal health, and is a critical point in a production system^10^.

Among the most studied immune nutrients are: conditionally-essential amino acids such as arginine and glutamine, which may become essential in stress situations; vitamins E and C, important antioxidants that prevent the aggressive effects of oxidative stress and help to preserve the proper functioning of the immunity; fatty acids that participate in the synthesis of inflammatory mediators, such as leukotrienes, prostaglandins, and thromboxanes, interceding in the immune system response^11^.

The use of immune nutrients such as fatty acids to modify inflammatory and immunologic responses has become of increasing interest both in animal and human health. It is because PUFA such as n-3 modulate immune system functions and thereby decrease the severity of inflammatory disorders^12^.

It is well documented that sterol regulatory element-binding protein 2 (*SREBP2*) preferentially activates genes involved in cholesterol biosynthesis and homeostasis^13–15^. The SREBPs modulate the transcription of genes encoding enzymes for FA synthesis and uptake, including fatty acid synthase *(FAS)*, acetyl CoA carboxylase (*ACC*), and stearoyl CoA desaturase-1 *(SCD1),* and lipoprotein lipase (*LPL*). Furthermore, *SREBP* has been associated with immune responses^16^, primarily because the sterols mediate the *SREBP* effects on immune function by altering membrane lipid composition, thus affecting signaling, stress responses, or binding to specific cellular receptors^17^.

The liver is a target tissue for FA-regulated gene expression and there is evidence that the PUFA are the principal FA regulating liver lipogenic gene expression^18,19^. The pig is an ideal animal model for investigating the effects of feeding different levels of soybean oil on liver transcriptome profiling and metabolic diseases that occurs also in humans. Pigs and humans show similarities in their anatomy, morphology, metabolism, and physiology, which indicates that the pigs are an important animal model in studies of metabolic diseases in humans such as obesity, atherosclerosis, diabetes, cancer, neurological, cardiopulmonary, and infectious diseases^20,21^. It was hypothesized that increasing the inclusion of soybean oil at different levels in the diets of growing-finishing pigs would modulate the liver gene expression profile and point out the main TF involved in fatty acid biological processes. Therefore, we have used the pig model to investigate key TF involved in lipid metabolism and immune response linked to differentially expressed genes (DEG), which were identified from liver tissue of immunocastrated male pigs fed either 1.5 or 3.0% added dietary soybean oil.

## Results

The blood biochemical parameters, body weight, muscle and liver fat content (ether extract), and the fatty acid composition of the liver of the animals fed with diets containing different levels of soybean oil (1.5% SOY1.5 vs 3.0% SOY3.0) were shown in the Supplementary Table S1. Among the blood biochemical parameters evaluated the albumin (g/dL), triglycerides (mg/dL), and very low-density lipoprotein (VLDL mg/dL) were statistically different (p < 0.05) between the two groups of diet, where the animals from SOY1.5 presented higher values of these parameters. The saturated (SFA), monounsaturated (MUFA), PUFA, and their ratio were statistically different (p < 0.05) between the groups, where we can observe a higher proportion of the sum of MUFA and PUFA and omega 3 (n-3) in the SOY3.0 group of diet. The same group presents lower triglycerides and VLDL in the blood. However, the animals did not present differences in the body weight, and subcutaneous, intramuscular, and liver fat deposition between the diets (Supplementary Table S1).

The RNA-Seq data from liver tissue of 35 pigs fed diets containing different levels of soybean oil (SOY1.5 n = 17 and SOY3.0 n = 18) was used for further analysis, once one of the samples presented a RIN value below the threshold. The mapping analysis statistic showed that 78% on average of total paired reads generated in this study were aligned against the *Sus scrofa* reference genome. After quality filtering, 19,250 genes were considered for differential gene expression analysis between the SOY1.5 and SOY3.0 groups. A total of 281 DEG (log2fold-change ≥ 1 or ≤ − 1; FDR-corrected p-value < 0.1) were identified, in which 129 were down-regulated (log2FC ranging from − 3.0 to − 0.20) and 152 were up-regulated (log2FC ranging from 4.8 to 0.24) in SOY1.5 group (Supplementary Table S2). The functional analysis from the list of down and up-regulated DEG in SOY1.5 by PANTHER applying the overrepresentation test showed no enrichment for GO terms from up-regulated DEG. However, we observed GO terms associated (p-value < 0.05) with Biological Processes from the list of down-regulated DEG, such as glucose metabolic process (GO:0006006), negative regulation of endopeptidase activity (GO:0010951), hexose metabolic process (GO:0019318), and others (Supplementary Table S3). The functional analysis detected important TF associated with lipid metabolisms, such as lipogenesis and adipogenesis, lipid homeostasis, and immune response (Supplementary Table S4).

**Figure 1:**
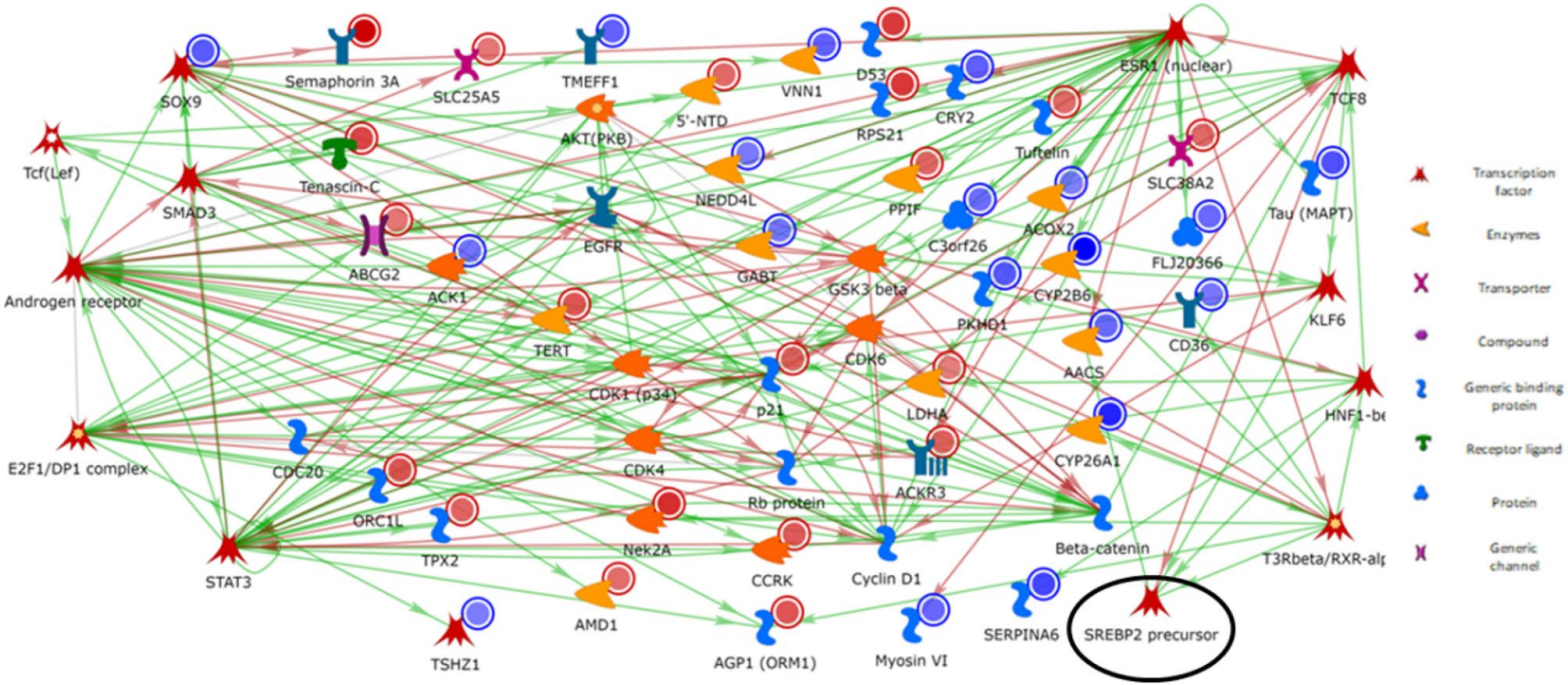
The network was created with MetaCore basic analysis networks (transcription factor) algorithm using DEG list (FDR < 0.1) from liver tissue of immunocastrated male pigs fed diets containing different levels of soybean oil (SOY1.5: 1.5% and SOY3.0: 3.0% soybean oil). The green lines represent the activation of other genes, whereas the red lines represent an inactivation. In the blue circles, genes down-regulated and up-regulated are highlighted with red circles. The SREBP2 precursor is highlighted with a black circle. Image created by MetaCore (Clarivate Analytics) [https://portal.genego.com/].

The genes and TF, including *CK1, AKT2, Beta-catenin, CDK6, Cyclin D1, EGFR, p21*, and *Rb protein*, are displayed in Supplementary Table S5. Both mechanisms of activation and inactivation of other genes and TF are shown in Fig. 1. The genes and TF, such as *Beta-catenina*,* EGFR*, and* ESR1*, lead to the primary activation of transcription (Supplementary Table S5). The genes *ACK1*,* AKT*,* CDK1*, and *CDK4*, cause phosphorylation effects with activation and inhibition mechanisms. From these, the TF, such as *TSHZ1* and *SOX9*, were also DEG with − 0.45 log2FC and − 1.02 log2FC, respectively.

The *RXRA* exhibited transcriptional regulatory response on some DEG, including *CYP25A1*, which codes for a cytochrome P450 enzyme superfamily and is related to the synthesis of steroids, cholesterol, and other lipids involved in activation mechanism^22^. Our results highlighted that *RXRA* could be involved in the regulation of other DEG, such as *HNF1-beta, ORM1, Nek2A, CD36,* and *CYP2B6*. Other TF are related to DEG, including *ESR1*, with binding, phosphorylate, and transcriptional regulate effects combined, as well as a co-regulatory effect with the TFs *SOX9* and *TCF8*.

**Figure 2:**
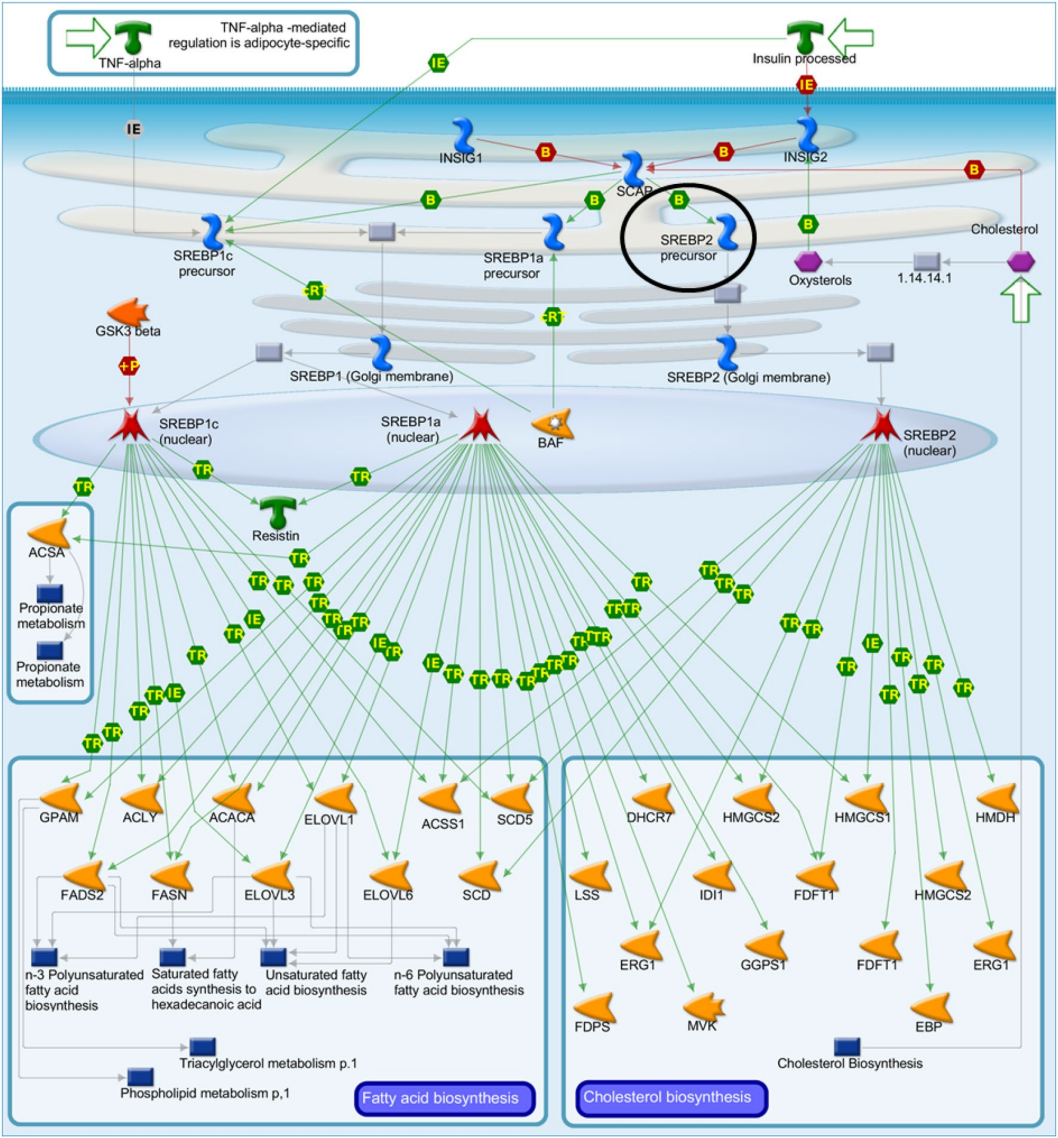
SCAP/SREBP transcriptional control of cholesterol and fatty acid biosynthesis pathway map. Image created by MetaCore (Clarivate Analytics) [https://portal.genego.com/]. Activation is indicated by green arrows, inhibition is indicated by red arrows, and unspecific relationship is indicated by gray arrows. The SREBP2 precursor is highlighted with a black circle. Nodes represent GeneGo Network objects (genes or gene complexes). For more information, see https://portal.genego.com/legends/MetaCoreQuickReferenceGuide.pdf.

**Figure 3:**
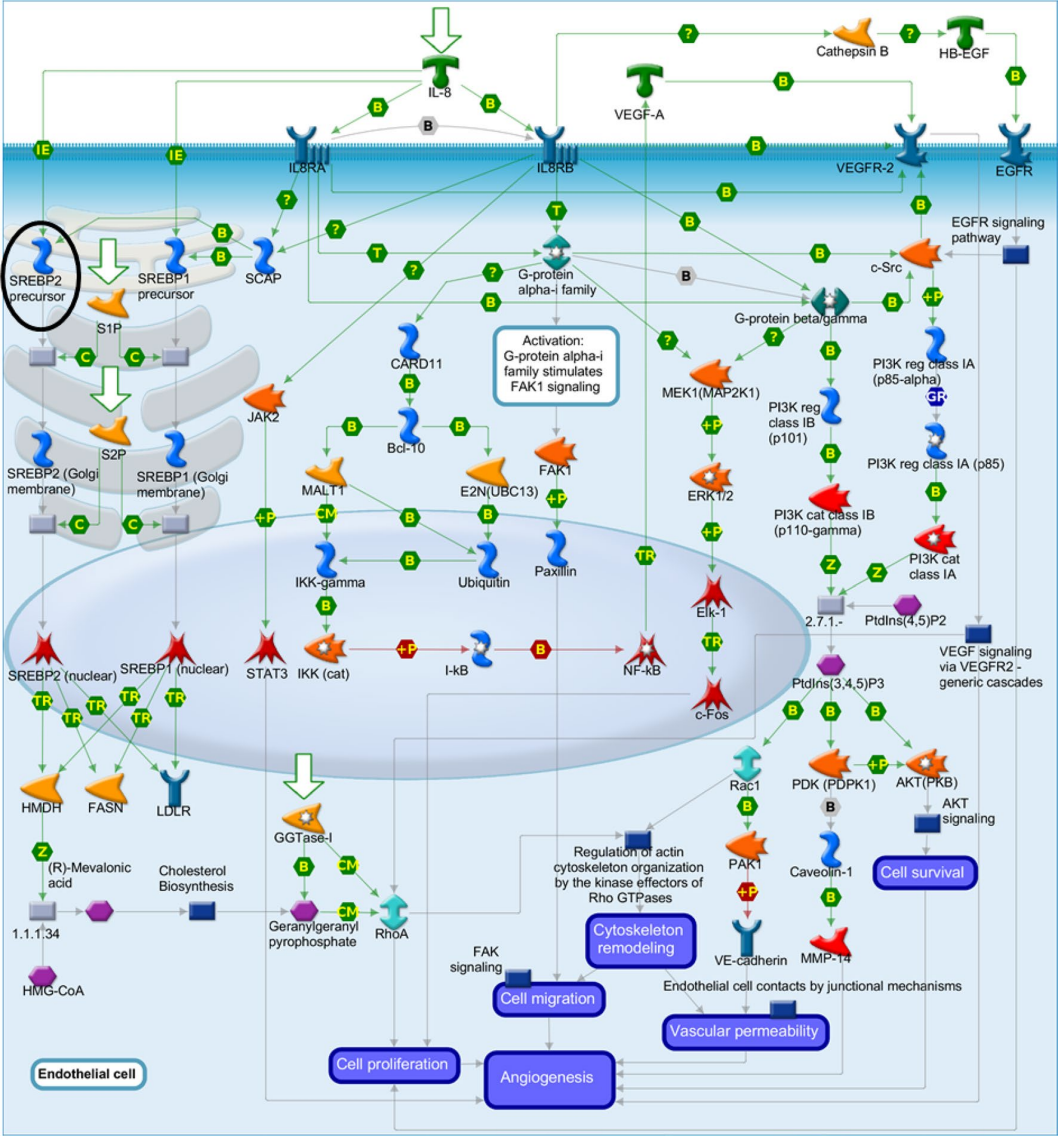
Development role of interleukin-8 (IL-8) in angiogenesis pathway map. Image created by MetaCore (Clarivate Analytics) [https://portal.genego.com/]. Activation is indicated by green arrows, inhibition is indicated by red arrows, and unspecific relationship is indicated by gray arrows. The SREBP2 precursor is highlighted with a black circle. Nodes represent GeneGo Network objects (genes or gene complexes). For more information, see https://portal.genego.com/legends/MetaCoreQuickReferenceGuide.pdf.

Based on the interactions observed in the network (Fig. 1), *SREBP2 precursor* is one of the key genes involved in the control of cholesterol and FA biosynthesis. This TF takes part in “SCAP/SREBP transcriptional control of cholesterol and FA biosynthesis”. The activation of *SREBP2 precursor* in sterol regulatory element-binding protein cleavage-activating protein (*SCAP*) is vital for targeting genes for FA and cholesterol biosynthesis (Fig. 2). The *SREBP2 precursor* was enriched in MetaCore analysis and undergoes transcriptional regulation via inhibition mechanisms by *TCF8*. The *SREBP2 precursor* is activated by *KLF6*,* T3Rbeta/RXR-alpha*, and *HNF1-beta*. Furthermore, *SREBP2* directly activates acetoacetyl-CoA synthetase (*AACS*) and was down-regulated in the SOY1.5 group compared to SOY3.0 group (− 0.75log2FC). Additionally, *SREBP2 precursor* may be involved in the angiogenesis via interleukin-8 (IL-8), considering IL-8 acts as a key mediator for inflammatory responses linked to angiogenesis^22^ (Fig. 3).

## Discussion

In this study, we observed that the increased level of soybean oil, an important source of MUFA, PUFA, and n-3 fatty acids, in the diet of animal models for metabolic diseases in humans such as type 2 diabetes, obesity, and coronary diseases, increased the proportion of these FA in the liver tissue. It also demonstrated a lower level of triglycerides and VLDL in the blood of the animals (SOY3.0), which did not affect the body weight and fat deposition^23^.

The transcriptome profile analysis identifies network connections, TF, and the behavior of interrelated genes in liver tissue in response to the increased levels of soybean oil in the diet of growing-finishing pigs. The enriched network showed that the DEG are involved in key regulatory mechanisms and soybean oil may alter the behavior of TF, such as inhibition of canonical *Wnt* signaling, adipogenic/lipogenic regulation, cholesterol metabolism, and others.

The *RXRA* was one of the TF detected herein, being fundamental for metabolism, development, differentiation, proliferation, and cell death. Moreover, RXRA acts on gene expression regulation and shows receptor behavior for FA and oxysterols^22^. According to Inoue et al.^24^ *RXRA* is related to hepatic triglyceride metabolism, in addition to be a transcriptional target of *PPAR*. The *RXRA* may display a transcriptional regulatory effect by the activation of DEG found in our study between SOY1.5 and SOY3.0 groups, such as the *CD36*, which was down-regulated in the SOY1.5 group.

It has been reported that the levels of the *CD36* protein are increased in nonalcoholic fatty liver disease (NAFLD) patients, which is associated with dyslipidemia, obesity, and type 2 diabetes^25^. Moreover, *CD36* gene is involved in liver steatoses^26^, with previous studies indicating an increase in liver steatoses in mice induced by a fat-rich diet^24^. In addition to the free fatty acid (FFA) transporter, *CD36* has an essential role in adipogenesis, mostly due to its high FA affinity^26^. The *CD36* deletion in mice resulted in the elimination of the LXR agonistic effect on the triglycerides and FFA in the liver and surrounding tissues^26^. Zhou et al*.*^26^ suggested that the *CD36* induction is related to an increase in *PPARγ* activation by the steatoses rather than an increase in FFA capitation. Our findings demonstrated a decrease in hepatic *CD36* expression in pigs fed SOY1.5 diet, suggesting that dietary inclusion of soybean oil altered *CD36* expression levels.

Another DEG with an activation interaction through RXRA transcriptional regulation was *CYP26A1*, a gene involved in lipid metabolism, which was negatively regulated in the SOY1.5 group. The *CYP26A1* alters fat deposition^27^ and takes part in the *CYP450* family, thus exhibiting a fundamental role in catalytic metabolic reactions and unsaturated FA oxidation^22^.

The *ESR1* TF shows a vital role in hepatic lipid and carbohydrate metabolism and is involved in the liver response to estrogen mediation^28^. Evaluating the changes in mice liver gene expression after the loss of the *ESR1* transcription regulation, Khristi et al.^28^ demonstrated a pattern of sex-related corporal body weight gain in females. While females became obese, the males do not even reach the normal corporal body weight, thus emphasizing the *ESR1* importance on lipid and carbohydrate metabolisms. Nuthikattu et al.^29^ identified the *ESR1* TF as a DEG modulated by the Western diet when comparing the gene expression between female mice groups with a deficiency of low-density lipoprotein receptors in the hippocampus microvessels fed either a control or occidental diet. According to Wiȩckowska-Gacek et al.^30^ occidental diet may cause metabolic syndromes and diseases due to several factors, including the low consumption of unsaturated FA. The *ESR1* TF (nuclear) participates in several DEG-related mechanisms and effects in our study, inhibiting the *RXRA* TF and the *NED4L* DEG and activating other genes such as *MAPT*, *ABT*, and *ACOX2.*

Another important connection factor is the *EGFR*. Considering the phosphorylation effect, *EGFR* acts as an activation mechanism in *ESR1*. The *EGFR* gene signaling may stimulate lipid metabolism; however, when overexpressed, this gene is linked to several types of cancer, including lung cancer^22^. In liver hepatocytes of adult mice, *EGFR* is more pronounced and has a critical function related to the liver repair and regeneration^31^. Furthermore, a novel FA synthesis is involved in EGFR activation, and in cells that store and secrete lipids, lipid metabolism may be stimulated by *EGFR* signaling^32^. In our study, *EGFR* appears to be involved in the mechanisms of phosphorylation to *AKT1* with an activation effect. In contrast, beta-catenin effect is of inhibition by the phosphorylation mechanism. Furthermore, the network interaction demonstrated that *ESR1* inhibits the SRY-box transcription factor 9 (*SOX*9).

The *SOX9* was a DEG up-regulated in the liver of pigs fed SOY1.5 diet compared to those fed SOY3.0 diet, and relevant to NAFLD development^33^. The authors suggested that *SOX9* may be considered a biomarker for NAFLD because it is involved in liver metabolic diseases since NAFLD pathology involves both molecular pathways and cellular alterations^33^. According to Stelzer et al.^22^ *FOXO* (*FOXO1* and* FOXO3*) promotes *SOX9* expression when the lipid levels are low, which is in agreement with our findings, given that *SOX9* expression was down-regulated in the SOY1.5 group. Moreover, SOX9 shows transcriptional regulation effects on the inhibition mechanism of *HNF1-beta, Semaphorin 3A*, and *CDK1* (p34), whereas activation effects were observed for *ABCG2*,* VNN1*,* RXRA*, and *KLF6* genes.

In the current study, we also highlighted the *TCF8/ZEB1* TF, which represses the expression of several genes, such as the interleukin-2 (IL-2), since *TCF8/ZEB1*, is involved in the up-regulation of neuronal differentiation^22^. The *TCF8/ZEB1* has an inhibitory effect on myosin VI, which was down-regulated (− 0.802 log2foldchange) in SOY1.5 group. Evaluating obesity/anti-obesity genes in parametrial fat cells from mutation or polymorphism in knockout *TCF8/ZEB1* mice, Saykally et al.^34^ demonstrated an association of *TCF8/ZEB1* with glucose uptake and adipose tissue accumulation.

The *SREBP* and TF regulate cholesterol, and along with the liver X receptor (*LXR*) family integrate cholesterol homeostasis with FA metabolism^35^. Furthermore, *SREBP* and *LXR* may be involved in the regulation of genes involved in immune system cells^35^. Herein, we found *SREBP2* precursor related to some DEG, indicating the involvement of our DEG in important regulations, such as cholesterol metabolism and other essential mechanisms in the liver. This TF acts primarily by activating genes encoding key enzymes of the lipid and cholesterol biosynthetic pathway^36^. In relation to the *SREBP* processing inhibition capacity, long-chain FA, such as eicosapentaenoic acid (EPA), docosahexaenoic acid (DHA), and arachidonic acid (AA), have a larger inhibition capacity in comparison to PUFA with smaller chains, as C18:1, C18:2, and saturated FA practically do not have effects over the *SREBP* processing^37^. The study of Horton et al.^38^ using transgenic mice expressing a dominant positive truncated form of *SREBP2* demonstrated that *SREBP2* is an activator of cholesterol biosynthesis, with stimulatory effects on genes associated with FA, and in equilibrium with regulatory proteins that limit FA biosynthesis.

In addition, *SREBP* plays a relevant role in the steroid metabolites, FA cell differentiation, and proliferation of T cells and natural killer (NK) cells. As reported by Kusnadi et al*.*^16^ *SREBP* are essential in factor-induced macrophage stimulation of tumor necrosis. The *SREBP* is associated with the activation of the immune response via the sterol metabolites from the cholesterol pathway involved in T cell proliferation^16^. Using mice cells induced by interleukin-2 (IL-2) and IL-12, Assman et al*.*^39^ reported that NK cells utilized glucose for amino acid and FA biosynthesis at 18 h post-stimulation^39^. This incorporation has been associated with the upregulation of SREBP signaling pathways, in which their target genes encode essential molecules for de novo synthesis of FA. Furthermore, *SREBP* may be involved in IL-8 pathway, a relevant pro-inflammatory cytokine in the modulation of the angiogenic process^40^. The PUFA may act in the angiogenesis through LOX enzymes, which lead to the formation of leukotrienes^41^.

Hepatic lipid and insulin induce *SREBP*-related adipogenesis, whereas *SREBP* promotes inflammatory responses, thus contributing to lipid metabolism and immune responses in the macrophages^41^. The effect of SREBP depends on a variety of environmental signals, including nutritional and inflammatory factors that occur via insulin signaling^42^. There is evidence that liver-specific knockout of the SREBP transport protein SCAP inhibited the activity of *SREBP* isoforms and prevents steatosis in mice fed high-fat diet^43^.

The *AACS* was identified as participating in the network and may be related to the mechanism of transcription regulation and the activation effect by the *SREBP2 precursor* TF when pigs were fed the SOY1.5 diet. The *AACS* plays an important role in the activation of acetoacetate to acetoacetyl-CoA, and hence may be related to the use of ketone body for FA synthesis during adipose tissue development^44^. The highest expression level of this gene may be obtained in brown and white adipose tissue^22^. The AACS is an acetoacetate-specific ligase. In mice, this gene is regulated in vivo in the liver tissue when induced by hypocholesterolemic agents^44^. Hasegawa et al*.*^44^ studied ketone bodies produced and released in the liver to produce energy in extra liver tissues along with *AACS,* which could be coordinated by SREBP2.

All DEG identified in the network have relevant functions and relationships with essential mechanisms in the liver. Cholesterol is crucial to the cell membrane, thus contributing to fluidity and permeability, participating in both membrane trafficking and transmembrane signaling^43^. Moreover, cholesterol is indirectly related to the control of most biological functions that occur and are facilitated in the membrane. Disturbances of lipid and cholesterol metabolism in the cell are related to diseases, such as cardiovascular and metabolic disorders^43^.

Our study corroborates the findings in the literature, in which FA modulate gene expression by key transcription factors in biological processes. However, studies are still limited to pigs and new approaches are needed, this study is the first work that explores the gene network to identify key TF involved in lipid metabolism and inflammatory response by transcriptome analysis from liver samples. The results found, in general, mainly related to differences found regarding the inclusion of soybean oil at different levels, in which the SOY1.5 group showed greater deposition of MUFA and PUFA in the liver compared to SOY3.0, in addition to the difference between albumin and triglycerides. This directly impacts functions associated with the lipid metabolism pathway, such as lipogenesis and adipogenesis, lipid homeostasis, and immune response both negatively and positively as seen with down-regulated and up-regulated DEG.

The liver, as a regulatory and central organ, controls lipid homeostasis through biochemical, cellular, and signaling pathways, endocrine activity, detoxification, and immunomodulation^42,45^. The detoxifying function is mainly for hazard product degradation and involves β-amyloid peptides (circulating Aβ). Furthermore, some factors such as insulin resistance hinder the detoxification process, leading to an overall increase in the Aβ level^30^. One of the first steps in AD progression refers to the appearance of pathological Aβ peptides. The Aβ peptides are related to MAPT deposits, thus causing intracellular tangles and plaque formation in blood vessels. Several physiological mechanisms in the liver regarding the Aβ removal and degradation may be related to AD progression^30^.

## Methods

All animal procedures were approved by the Animal Care and Use Committee of Luiz de Queiroz College of Agriculture (University of São Paulo, Piracicaba, Brazil, protocol number: 2018.5.1787.11.6 and number CEUA 2018-28) and followed ethical principles in animal research, according to the Guide for the Care and Use of Agricultural Animals in Agricultural Research and Teaching^46^. This study was carried out in compliance with the ARRIVE guidelines.

### Animals and experimental diets

The current study used data from ALMEI*DA *et al.^23^ and SILVA et al*.*^47^. Briefly, a total of 36 immunocastrated and halothane homozygous-negative (NN) male pigs (*Large White*) were used in a 98-day feeding trial. Pigs were housed in an all-in/all-out double-curtain-sided building with partially slatted concrete floor pens. Immunocastration of intact males was performed by administering two 2-mL doses of Vivax^®^ (Pfizer Animal Health, Parkville, Australia) at 127 and 141 days of age. The animals used herein were from a population genotyped for the halothane mutation (RYR1 gene)^48^.

Pigs were blocked by initial body weight (28.44 ± 2.95 kg) and assigned to one of two dietary treatments: 1.5% soybean oil (SOY1.5) or 3.0% of soybean oil (SOY3.0). The levels of soybean oil to be tested in this study were decided based on the usual nutritional program in pig production, which is 1.5% of soybean oil. Each treatment had six pens containing three pigs. Diets were formulated to meet or exceed the nutritional requirements of growing-finishing pigs, as defined by Rostagno^49^. Feed treatments consisted of corn-soybean meal in the growing period, while diets in the finishing period were added with SOY1.5 or SOY3.0. Pigs were fed in a 5-phase feeding program that lasted from day 0 to 21 for grower I; days 21 to 42 for grower II; days 42 to 56 for finisher I; days 56 to 63 for finisher II; days 63 to 70 for finisher III; and days 70 to 98 for finisher IV^23^. All pigs had ad libitum access to feed and water throughout the experimental period. Individual pig Body weight was measured on days 0, 21, 42, 56, 63, 70, and 98.

At the end of the 98-day study, pigs with an average final body weight of 133.9 ± 9.4 kg (169 days old) were slaughtered by electrical stunning followed by exsanguination, according to the industry standards and Brazilian legislation, after a 16-h rest period. After slaughter, liver tissue samples were collected, snap-frozen in liquid nitrogen, and stored at -80ºC until analysis.

### Blood biochemical parameters and fatty acid profile of liver

Blood was sampled from the jugular vein four days before the slaughter and immediately transferred into non-anticoagulant vacuum tubes (Becton Dickinson Vacutainer Systems, Franklin Lakes, NJ, USA). Then, the samples were stored at room temperature for 2 h, subsequently centrifuged at 3000×*g* for 10 min to obtain serum, and stored in duplicated 1.5-mL tubes at − 80 °C. Serum lipid and biochemistries were analyzed by the Mindray, BS120 (Guangdong, China) in the Pathology Laboratory at the University of São Paulo, Pirassununga, SP, Brazil. Blood serum glucose content was quantified by the colorimetric enzymatic method according to Trinder^50^ using commercial kits, following the use recommendations proposed by the manufacturer. The quantification of total cholesterol and fractions was also performed by the enzymatic-colorimetric method, but by selective precipitation. This procedure was performed using commercial kits, according to the manufacturer's instructions. The analysis for the determination of total proteins was performed using commercial kits, following the used protocol proposed by the manufacturer, using the Biureto method with some modifications^51^. The composition of FA was previously investigated by our group and described in ALMEIDA et al*.*^23^ and SILVA et al.^47^. Fatty acid composition analyses: Ether extract was obtained from 5 g of skeletal muscle (*Longissimus lomborum*) and liver using the Soxhlet method according to AOAC (Method 963.15)^52^. For fatty acid profile determination, total lipid was isolated from 100 g of skeletal muscle and 25 g of the liver; following the cold extraction method proposed by Bligh and Dyer^53^ and methylated according to the procedure outlined by AOCS (Method AM 5-04)^54^.

### RNA extraction, libraries, and sequencing

Total RNA was extracted from 30 mg of frozen liver samples using RNeasy^®^ Mini Kit (QIAGEN, Hilden, Germany) following the manufacturer’s guidelines. The RNA integrity was verified by Bioanalyzer 2100 (Agilent, Santa Clara, CA, USA) and only samples with RIN score > 7.8 were used. A total of 2 μg of total RNA from each sample was used for library preparation according to the protocol described in the TruSeq RNA Sample Preparation kit v2 guide (Illumina, San Diego, CA)^55^. The average insert size of the libraries was estimated using the Agilent Bioanalyzer 2100 (Agilent, Santa Clara, CA, USA) and quantified using quantitative PCR with the KAPA Library Quantification kit (KAPA Biosystems, Foster City, CA, USA). Then, samples were diluted and pooled (three pools of six samples each). Five lanes with 36 pooled samples were performed in two sequencing flowcell, using the TruSeq PE Cluster kit v4-cBot-HS kit (Illumina, San Diego, CA, USA), clustered, and sequenced using HiSeq2500 ultra-high-throughput sequencing system (Illumina, San Diego, CA, USA) with the TruSeq SBS Kit v4-HS (200 cycles), according to manufacturer instructions^55^. The sequencing analyses were performed at the Genomics Center at ESALQ, Piracicaba, São Paulo, Brazil.

### Data analysis, differentially expressed genes, and functional enrichment analysis

The RNA-Sequencing (RNA-Seq) data quality was checked using the FastQC, v. 0.11.8 software [http://www.bioinformatics.bbsrc.ac.uk/projects/fastqc/]. Adapters and bases with low PHRED scores were removed using the Trim Galore v. 0.6.5. Reads with a minimum length of 70 bases were aligned and mapped to the reference pig genome (*Sus Scrofa* 11.1) using the assembly available at Ensembl Release 102 [http://www.ensembl.org/Sus_scrofa/Info/Index]. Alignment and mapping were performed using the STAR v. 2.7.6a.

All DEG were compared between treatments (SOY1.5 *vs* SOY3.0) from liver tissue, which were identified using the DESeq2 package available at Bioconductor open-source software for bioinformatics^56^, using a multi-factor design. Before statistical analysis, the read count data was filtered as follows: (i) genes with zero counts for all samples, that is, unexpressed genes, (ii) genes with less than 1 read per sample on average were removed (very lowly expressed); (iii) genes that were not present in at least 50% of the samples were removed. For statistical analysis of transcript abundance, sire was fit as a factor in the multi-factor model. To control false discovery, a false discovery rate (FDR) was set at 10%^55^ with FDR determined using the Benjamini–Hochberg^57^ methodology. Significance differences were set at log2fold-change ≥ 1 or ≤ − 1; FDR-corrected p-value < 0.1.

The functional enrichment analysis of DEG was performed to obtain comparative networks by analyzing the single network (TF) using a standard parameter of MetaCore software (Clarivate Analytics) v. 21.3 build 70600^58^, which were filtered for liver tissue and *Homo sapiens* species dataset to show activating and inhibiting effects. The overexpression test from the list of up and down-regulated DEG was performed by Panther v.17 (http://www.pantherdb.org/)^59^ to identify the GO terms related to Biological Processes. The *Sus scrofa* genome reference was used as background.

For the functional analysis to identify important TF related to lipid homeostasis and immune response, the construction of the gene network by the TF mechanism was performed. For each TF of the master list, the algorithm generated a subnetwork with all the shortest paths to which the TF from the nearest recipient with direct ligands on the list. The construction favors networks, in which the terminal nodes (recipient targets) of transcription-regulated pathways on the original gene list provide a TF-specific network on the gene list.

## Conclusions

In this study, we identified the transcription factors RXRA, EGFR, and SREBP2 precursor as key transcription factors linked to lipid homeostasis and inflammatory response from liver transcriptome profile of animal models for human metabolic diseases fed with different levels of dietary soybean oil. The results described herein could contribute to the nutrigenomics research field that aims to elucidate dietary interventions in animal and human health, as well as to drive food technology and science.

## Supplementary Information

Supplementary Information 1.

Supplementary Information 2.

Supplementary Information 3.

Supplementary Information 4.

## Data Availability

The dataset supporting the conclusions of this article is available in the European Nucleotide Archive (ENA) repository (EMBL-EBI), under accession PRJEB50513 [http://www.ebi.ac.uk/ena/data/view/PRJEB50513].
