# Supplementary material for: Effect of dietary soybean oil inclusion on liver-related transcription factors in a pig model for metabolic diseases
Source: Sci Rep. 2022 Jun 20;12:10318. doi: 10.1038/s41598-022-14069-1 (PMC9209463; doi:10.1038/s41598-022-14069-1)
Supplement: Supplementary file 4 — Supplementary Information 4. [file 41598_2022_14069_MOESM4_ESM.docx]

**Table S1.** Effect of the diets on blood biochemical parameters and fatty acid profile of liver from immunocastrated male pigs fed diets containing different levels of soybean oil (SOY1.5: 1.5% and SOY3.0: 3.0% soybean oil).

| Variable | Treatments | | Pooled SEM¹ | P-value |
| --- | --- | --- | --- | --- |
|  | SOY1.5 | SOY3.0 |  |  |
| Glucose(mg/dL) | 81.85 | 82.85 | 4.17 | 0.81 |
| Aspartataminotransferase (U/L) | 40.35 | 38.13 | 2.42 | 0.37 |
| TotalProteins (g/dL) | 6.69 | 6.47 | 0.13 | 0.11 |
| Albumin(g/dL) | 3.70^a^ | 3.46^b^ | 0.11 | <0.05 |
| Globulin(g/dL) | 2.99 | 2.99 | 0.12 | 0.98 |
| Triglycerides(mg/dL) | 44.48^a^ | 35.70^b^ | 4 | <0.05 |
| Cholesterol(mg/dL) | 99.34 | 96.55 | 4.58 | 0.55 |
| HDL(mg/dL) | 44.34 | 43.72 | 2.06 | 0.77 |
| LDL(mg/dL) | 45.99 | 45.71 | 2.91 | 0.92 |
| VLDL(mg/dL) | 9.00^a^ | 7.13^b^ | 0.81 | <0.05 |
| Body weight (kg) | 130.61 | 131.17 | 3.0430 | 0.86 |
| Intramuscular fat (%) | 1.94 | 2.63 | 0.35 | 0.06 |
| Liver fat (%) | 2.6750 | 3.1878 | 0.6520 | 0.44 |
| Total SFA (%) | 46.69 | 45.24 | 1.03 | 0.31 |
| Total MUFA (%) | 22.01^a^ | 28.78^b^ | 1.04 | <0.01 |
| Total PUFA (%) | 30.79^a^ | 26.06^b^ | 0.55 | <0.01 |
| Total n-3 PUFA (%)^2^ | 3.75^a^ | 2.42^b^ | 0.37 | <0.01 |
| Total n-6 PUFA (%)^3^ | 27.02^a^ | 23.64^b^ | 0.67 | <0.01 |
| PUFA:SFA ratio (%)^4^ | 0.67^a^ | 0.58^b^ | 0.02 | <0.01 |
| n-6:n-3 PUFA ratio^5^ | 8.51^a^ | 9.9^b^ | 0.5 | <0.05 |
| Atherogenic index^6^ | 0,45^a^ | 0.49^b^ | 0.18 | <0.01 |

^1^ SEM = standard error of the least square means.

^2^ Total n-3 PUFA = {[C18:3 n-3] + [C20:5 n-3] + [C22:6 n-3]}.

^3^ Total n-6 PUFA = C18:2 n-6.

^5^4PUFA:SFA ratio = total PUFA/total SFA.

^5^ Σ n-6/Σ n-3 PUFA ratio.

^6^ Atherogenic index = (4 × [C14:0]) + (C16:0)/(total MUFA] + [total

PUFA]) [1], where brackets indicate concentrations.

a-b Within a row, values without a common superscript differ (P ≤ 0.05) or tended to differ (0.05 < P ≤ 0.10) using Student’s t test.

**Table S4.** Transcription factors associated identified from the functional annotation analysis by MetaCore (Clarivate Analytics) [<https://portal.genego.com/>] from the list of differentially expressed genes of liver tissue of immunocastrated male pigs fed with two different soybean oil proportions in the diet (SOY1.5: 1.5 % and SOY3.0: 3.0 % of soybean oil).

| **Transcription factor** | **Description** | **Reference** |
| --- | --- | --- |
| **T3Rbeta / RXR-alpha, RXRA**  retinoid X receptor alpha | Along with PPARα, it represses the transcriptional activity of HNF4α. | [10.1016/j.metabol.2021.154705](https://doi.org/10.1016/j.metabol.2021.154705) |
| ***HNF1-beta***  HNF1 homeobox B | It may be involved in the inhibition of canonical Wnt signaling as it competes with the binding of β-catenin/LEF complexes. It is also related to the regulation of liver, kidney, pancreas and other epithelial organs. | 10.1073 / pnas.1909452116 |
| ***E2F1 - E2F1/DP1 complex***  E2F transcription  factor 1 | Important in lipogenesis, it is also the progression of the cell cycle and induction of apoptosis in case of DNA damage. During the initial phase of adipogenesis, it can trigger PPARγ expression. When dysregulated, it activates cytoplasmic Ras/mitogen-activated protein (MAPK) signaling cascades. | [(1)10.1016/j.anndiagpath.2019.01.002 (2)10.1016/S1534-5807(02)00190-9](https://doi.org/10.1016/j.anndiagpath.2019.01.002) |
| ***ESR1 (nuclear)***  estrogen receptor 1 | It is crucial for liver lipid and carbohydrate metabolism. Its impaired function can lead to obesity and metabolic dysfunction. | [10.1016/j.mce.2019.04.005](https://doi.org/10.1016/j.mce.2019.04.005) |
| ***STAT3***  signal transducer and activator of transcription 3 | Fundamental in cell growth and apoptosis, it can be activated in response to various cytokines and growth factors, including EGF, IFNs, IL5, IL6, LIF, HGF, and BMP2. | Stelzer, G. et al. 2016 |
| [***KLF6***](https://portal.genego.com/cgi/regulation/regulation_info.cgi?id=-523566610)  Kruppel like factor 6 | Co-regulates lipid homeostasis. A study performed with KLF6 knockdown revealed pathways associated to lipid homeostasis (cholesterol and triacylglycerol biosynthesis) which are down-regulated genes, in addition to SREBF1 and SREBF2. | <https://www.nature.com/articles/s41467-019-09116-x> |
| ***SMAD3***  SMAD family member 3 | Activated in liver fibrosis. Involved in intracellular TGF-β signaling, body development, homeostasis and metabolism. | [10.1369/0022155415627681](https://doi.org/10.1369/0022155415627681) |
| ***SOX9***  SRY-box transcription factor 9 | FOXO transcription factors (FOXO1 and FOXO3) promote SOX9 expression when lipid levels are low and thus induce chondrogenic compromise, suppressing fatty acid oxidation. Furthermore, SOX9 may be related to inhibition of Wnt signaling. | [(1) Stelzer, G. et al. 2016 (2)10.1002 / dvdy.22046](https://doi.org/10.1016/j.anndiagpath.2019.01.002) |
| ***RXRA***  retinoid X receptor alpha | Related to adipogenic / lipogenic regulation. The SNPs ENHO, RXRA and LXRA were linked to epistatic interactions in dyslipidemia and myocardial infarction. | 10.1186 / s12881-018-0708-4 |
| ***TCF8***  zinc finger E-box binding homeobox 1 | It regulates endothelial invasion and may negatively regulate pathological angiogenesis. | [10.1016/j.bbrc.2008.12.101](https://doi.org/10.1016/j.bbrc.2008.12.101) |
| ***TSHZ1***  teashirt zinc finger homeobox 1 | It may be related to transcriptional regulation of the developmental processes. | Stelzer, G. et al. 2016 |
| ***TCF(LEF)***  lymphoid enhancer binding factor 1 | In the nucleus, it binds to β-catenin and can activate transcription of Wnt target genes. | 10.15252 / embj.201798873 |
| ***Androgen receptor*** | Steroid-hormone activated  transcription factor. | Stelzer, G. et al., (2016). |
| ***SREBP2* precursor**  sterol regulatory element binding transcription factor 2 | Related to cholesterol homeostasis by regulating the transcription of sterol-regulated genes. | Stelzer, G. et al., (2016). |

References

^1^Ulbricht, T. L. V. & Southgate, D. A. T. Coronary heart disease: seven dietary factors. *Lancet* **338**, (1991).
